# Erosion and deposition beneath the Subantarctic Front since the Early Oligocene

**DOI:** 10.1038/s41598-019-45815-7

**Published:** 2019-06-26

**Authors:** Uisdean Nicholson, Dorrik Stow

**Affiliations:** School of Energy, Geoscience, Infrastructure and Society, Heriot-Watt University, Edinburgh, EH14 4AS Scotland UK

**Keywords:** Sedimentology, Physical oceanography

## Abstract

The Antarctic Circumpolar Current (ACC) spills across the Falkland Plateau into the South Atlantic as a series of high-velocity jets. These currents are a driving force for global overturning circulation, and affect climate by modulating CO_2_ exchange between the atmosphere and ocean, but their timing of onset remains controversial. We present new evidence of strong currents associated with the Subantarctic Front (SAF) jet since the earliest Oligocene (~34 Ma) based on a widespread erosional surface on the Falkland Plateau, preserved below a 30,000 km^2^ contourite sand deposit. This is the largest such feature ever to be recognized, and provides the most robust constraint of the initiation of the SAF to date. By contrast, the South Falkland Slope Drift is dominated by contourite mud of Pleistocene-Recent age, substantially younger than previous estimates, indicating a significant decrease in long-term current strength at that time. As ACC strength is primarily a function of the position of the South-Westerly Winds, our data indicates that associated currents are likely to increase substantially in a warming world. Likely implications include increased upwelling and associated carbon flux from the deep ocean to the atmosphere, a positive feedback loop not included in most future projections of atmospheric CO_2_.

## Introduction

The Antarctic Circumpolar Current (ACC), the world’s largest moving body of water, is a wind-driven current that flows continuously around the Antarctic continent, transporting ~134 Sverdrups (Sv) of water^1^ and forming a pronounced oceanographic barrier between the cold waters surrounding Antarctica and the warmer waters of the Pacific, Atlantic and Indian oceans. The ACC is driven by surface shear stress induced by the South Westerly Winds (SWW), with surface currents capable of driving circulation of both intermediate and deep water masses. This wind-driven current system plays a critical role in the interconnected global ocean circulation and climate system, partly driving the Atlantic Meridional Overturning Circulation^2^, Agulhas leakage via the ‘warm-water route’ into the South Atlantic^3^, inducing upwelling and carbon exchange with the deep Southern Ocean^4,5^ and affecting ice-sheet stability in Antarctica^6^.

The main factor impacting the strength of the ACC is the alignment of the SWW with the Drake Passage gateway between South America and West Antarctica^7^, with wind belts having shifted substantially southward^8^ and increased in strength^9^ in the last few decades. Although this short-term (decadal) shift in wind strength has not yet been transmitted to the deep ocean in terms of changes in current strength^9^, it is clear that longer term trends do affect deep currents. Millennial-scale latitudinal migrations in the SWW appear to be related to out-of-phase warming and cooling conditions in the northern hemisphere, the so-called “bipolar seesaw” effect^10^, demonstrating the complex teleconnections between atmospheric CO_2_ content, warming and cooling conditions in both hemispheres, and global ocean circulation. Recent studies have shown that latitudinal shifts in SWW have had a critical effect on circulation and climate. These occurred in response to Dansgaard-Oeschger events during the most recent glacial period^11^ and in the transition from the last glacial to the current interglacial, when the ACC appears to have increased in intensity^12^.

However, despite their importance to palaeoceanographic and climate models, there are no substantial records of the longer-term changes in current strength associated with the ACC. Even the timing of onset of current activity remains disputed, with published estimates ranging from the Late Eocene to late Middle Miocene^13^. Long-term records are particularly important as the Earth warms to a pre-Pleistocene state, with CO_2_ levels and temperatures rapidly rising, wind-belts shifting polewards and a seasonal ice-free Arctic likely in the next few decades^14^. This would have an important impact on the strength of the currents associated with the ACC, and possible associated feedback mechanisms, including upwelling and CO_2_ exchange between the atmosphere and the deep ocean^5^.

**Figure 1:**
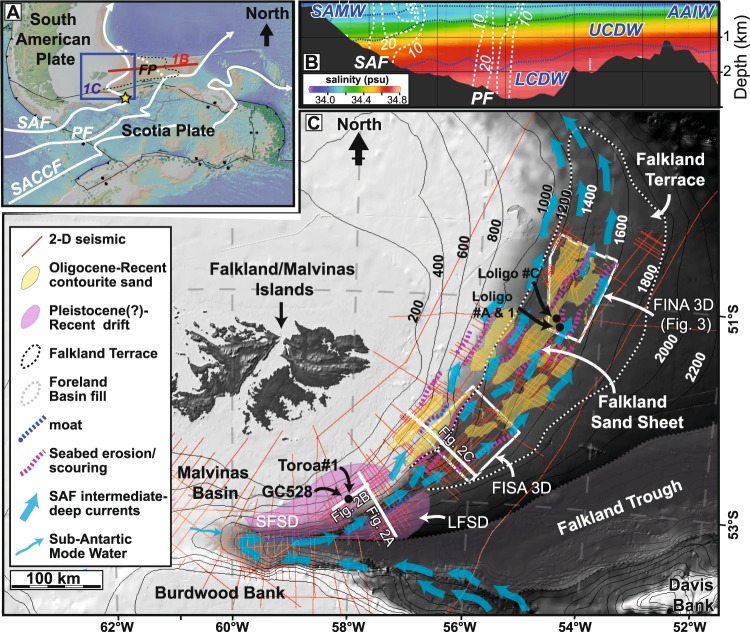
(**A**) Topographic features, plate tectonic and oceanographic setting of the Falkland Plateau (FP; outline in black dashed line), showing locations of main fronts of the Antarctic Circumpolar Current, including the Subantarctic Front (SAF), Polar Front (PF) and Southern Antarctic Circumpolar Current Front (SACCF)^15^. The yellow star shows the location of the 54–54 passage, where the SAF crosses the North Scotia Ridge between Burdwood Bank and Davis Bank. Image created using GeoMapApp. (**B**) Salinity and velocity data across the Falkland Plateau from the World Ocean Atlas, created using Ocean Data View software^48^. Blue dashed line shows the pycnoclines between water masses, including Subantarctic Mode Water (SAMW), Antarctic Intermediate Water (AAIW), Upper Circumpolar Deep Water (UCDW), and Lower Circumpolar Deep Water (LCDW). White dashed lines are velocity contours (in cm/s), measured using acoustic doppler current profilers on a parallel transect^17^, and show the location of the SAF and the PF. FT is the Falkland Terrace (**C**) Location map of the western Falkland Plateau showing depth contours (in metres), seismic and well data, and main sedimentary/geomorphological features associated with the SAF, including the main contourite drifts and the Falkland Terrace and the individual sand deposits that make up the Falkland Sand Sheet, based on the presence of high impedance seismic packages with distinct geomorphological features at the seafloor. SFSD – South Falkland Slope Drift; LFSD – Lower Falkland Slope Drift.

The principal exchange of water masses of the Southern Ocean with the South Atlantic Ocean, the ‘cold water route’, takes place across the North Scotia Ridge, which separates the Scotia Plate from the South American Plate, the latter including an E-W trending continental promontory known as the Falkland Plateau (Fig. 1A). The majority of the water of the ACC is transported across the ridge through a series of narrow passages, by high-velocity jets^15^, including the Subantarctic Front (SAF) through the 54–54 passage (52 Sv) between Burdwood Bank and Davis Bank, and the Polar Front (PF) through the Shag Rock Passage (48 Sv)^1^ (Fig. 1). Currents passing through these narrow passages are associated with the formation of meso-scale eddies^16^, where velocities are temporally elevated by the formation of vortices extending down to the seabed.

The SAF flows northward across the Falkland Plateau, with the core of the current approximately paralleling the 1600 m isobath^17^ (Fig. 1B). Elevated current velocities (>10 cm/s) extend from a depth of around 1400 m, close to the pycnocline between Antarctic Intermediate Water (AAIW) and Upper Circumpolar Deep Water (UCDW), to ~1800 m. The current therefore transfers water masses associated with Subantarctic Mode Water (SAMW), AAIW and UCDW. This current is associated with extensive contourite drifts, including the South Falkland Slope Drift (SFSD)^18^, which represent a valuable archive of current processes through time. A number of these drifts have been described by previous authors using multi-channel seismic data^18–20^ and in some cases penetrated by shallow (2–3 m) kasten cores^21^. A gravity core (GC528) acquired in 2011 penetrated the SFSD to a depth of 7 m, in close proximity to the Toroa#1 core that we use in this study. This well documented an increase in current strength at the end of the last glacial period, based on grain size and other proxies, but the record is limited to the Late Pleistocene (25 ka)^12^ due to the shallow depth of penetration.

The onset and evolution of the SAF was dependent on the opening and deepening of the Drake Passage, or precursor gateways^22–24^ between South America and Antarctica, as well as the formation of deep-water passages along the North Scotia Ridge^25^, an area where the tectonic history and palaeo-bathymetry remains poorly constrained. Previous estimates for the onset of deposition by bottom currents from the SFSD are based on proxy data, such as average sedimentation rates^18^ and long-distance seismic correlation^20^, both giving Early Miocene ages. Other contourite drifts in the Scotia Sea have also been used to infer the onset of ACC and changes in ACC current strength since the Miocene, but these are also based on limited core calibration, and are affected by Weddell Sea Deep Water formation as well as the ACC^26^.

We here use extensive 3D and 2D seismic reflection data and three recently acquired geotechnical boreholes from the Falkland Plateau to constrain the age and origin of the SFSD and the newly-identified Falkland Sand Sheet (FSS), in order to better constrain the dynamics of the ACC, and specifically currents associated with the SAF, through time. These new results provide robust confirmation for the Oligocene onset of strong intermediate-deep currents associated with ACC circulation and the Pleistocene transition to more weak-to-moderate current conditions, with clear implications for modelling ocean circulation in a world with pre-Pleistocene atmospheric conditions. Furthermore, we document, for the first time, the nature of the Falkland Terrace, which displays large-scale erosional features, and the extensive FSS, which formed beneath the SAF. This clearly demonstrates the significance of strong bottom currents in shaping slope architecture and in depositing large quantities of sand in deep water.

### Falkland Plateau slope morphology

**Figure 2:**
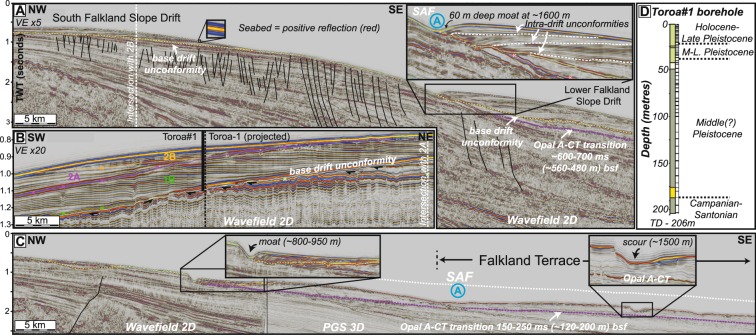
(**A**) Regional 2-D seismic line across the Falkland Plateau slope, across the South Falkland Slope Drift and Lower Falkland Slope Drift. All seismic sections are in the time domain (vertical axis is Two-Way Time) and have a ~5x vertical exaggeration (VE) unless otherwise stated. Blue, circled, letter A represents the core of the current flowing away from the reader. (**B**) Compressed (~20x VE) seismic section across the SFSD, with main horizons from Koenitz *et al.*^18^, and location of the Toroa#1 geotechnical borehole. Black arrows indicate erosional truncation of underlying seismic reflections at the base-drift unconformity. (**C**) Regional 2-D seismic line across the central Falkland Plateau, showing stepped/terraced morphology and erosional (and minor depositional) features associated with the SAF. The white dashed line indicates the possible seafloor height before erosion by the SAF. Seismic lines were originally acquired by Wavefield and PGS. (**D**) Lithologies and interpreted ages of the Toroa#1 borehole through the SFSD, acquired and analysed by BHP. Core recovery is shown by the black (sample) and white (core gap) barcode to the right of the lithology column. The key to the lithology is shown in Fig. 4 and detailed biostratigraphy is shown in Appendix A.

The slope of the southern and eastern Falkland Plateau has a complex morphology, with a number of terraces and locally mounded morphological features which are characteristic of margins dominated by bottom-current activity^27^. A number of pronounced changes in slope gradient and morphology, both down the slope and along the slope from south-east to north-east, are evident from bathymetric data (Fig. 1) and seismic data (Fig. 2).

The south-western slope is characterized by two extensive sediment drifts, with characteristic convex-up morphologies, separated by a slope-parallel erosional moat at around 1600–1700 m water depth (Fig. 2A). This moat separates the SFSD from another drift deposit, which we herein refer to as the Lower Falkland Slope Drift (LFSD). Both of these drifts sit above a prominent erosional unconformity (the base drift unconformity). Truncation of underlying seismic reflectors below the unconformity (Fig. 2B) indicates that there may be more than 100 ms of missing sequence in this location.

Along slope to the north-east, the slope flattens out into a broad terrace at water depths of ~1200–1800 m, which we refer to as the Falkland Terrace (Fig. 2C). The terrace forms part of a broader area of slope erosion, with evidence for along-slope erosion occurring up to water depths of 800 m, and locally even as shallow as 500 m (Fig. 1). There are a number of prominent moats evident in the seismic data, the shallowest of which is situated at around 800–950 m water depth (Fig. 2C), as well as other indications of sea-floor scouring along the main Falkland Terrace. In some locations, there are also depositional features, including minor drifts, some of which are associated with moats and other erosional features (Fig. 2C, inset). These coincide with areas of higher seabed amplitudes, with the larger acoustic impedance contrast indicating a distinct change in lithology at the seabed.

A prominent feature of the shallow subsurface across the Falkland Plateau is a ‘hard’ (increase in acoustic impedance) reflector, which is situated between 150–700 ms below the seabed, and which cross cuts stratigraphy (Fig. 2A,C). The discordant nature and seismic polarity of the reflection is consistent with an Opal A-CT transition^28^. This transition is one of a group of ‘bottom simulating reflectors’ (BSRs), and represents the diagenetic transformation of silica from a non-crystalline to a disordered crystalline form^29^. The primary control on this reaction is temperature, with other factors such as lithology, pore-fluid chemistry and time having a lesser influence^30^. The Opal A-CT transition is a non-reversible diagenetic process, with Opal remaining in a crystalline form even when temperature and pressure are reduced, thus it acts as a palaeothermometer, recording the maximum temperature experienced by the sediments.

The Opal A-CT transition in the south-western Falkland Plateau is situated at a maximum of around 700 ms TWT below the seabed (Fig. 2A), which is equivalent to around 560 m based on Loligo-1 velocity data (~1600 m/s). Using a geothermal gradient of 38.5 °C/km and a seabed temperature of 2 °C from Loligo-1, this is equivalent to a temperature of ~22 °C. This is at the lower bound (~20 °C) of temperatures derived from wells associated with clear geophysical expressions of Opal A-CT transitions elsewhere, most of which exceed 35–40 °C^28,31,32^. Opal A-CT transitions at significantly greater depths, with temperature greatly exceeding this value have also been reported in some instances, where the vertical (upward) migration of the diagenetic front has been arrested, or fossilized, for reasons that remain poorly understood^31,33^. However, empirical observations of shallower and lower temperature (~16–20 °C) transitions are rare^28^, and are only found in association with large-scale slide scars representing widespread seabed erosion

Under the Falkland Terrace, however, the Opal A-CT transition is significantly shallower than in the south-western Falkland Plateau region, typically around 250 ms (~200 m) and as little as 150 ms (~120 m) below the seabed, representing temperatures of ~10 and ~6 °C respectively. These temperatures are significantly lower than any experimental or empirical observations for the onset of the Opal A-CT transformation^28,32^. These anomalously low present-day temperatures suggest that subsurface temperatures are, or were in the past, higher in this area. In the case of the Falkland Terrace, assuming that there are no significant lateral changes in geothermal gradient, we interpret the anomalously shallow Opal A-CT transition to be a result of widespread seafloor erosion (~300 m, locally exceeding 400 m) across the Falkland Terrace region since the Opal A-CT transition initially formed. This area of maximum erosion is directly associated with the core of the SAF, suggesting that elevated bottom current velocities associated with the front are, or have been, strong enough to erode, entrain and transport substantial quantities of sediment.

### Contourite drifts

The SFSD is a plastered drift deposit that extends from ~500 m to 1500 m water depth, across an area of over 6600 km^2^ (Figs 1 and 2). The base of the drift is marked by a ~60 m deep, SW-NE trending erosional moat at 1600 m (Fig. 2A) separating it from the ~2000 km^2^ LFSD. The base of the LFSD is indistinct due to incomplete seismic coverage, but likely extends to the Falkland Trough, where a thick Neogene sequence is present^34^.

The SFSD was penetrated by the Toroa#1 geotechnical borehole, which extended to the base of the drift at the well location (Fig. 2B), with recovery rates of ~30% in the shallow 40 m and ~10% below that. This well penetrated a thick (175 m) sequence of Middle Pleistocene-Holocene mud, with minor silt layers, above a thin (10 m) clay-rich sand of indeterminate age (Fig. 2D). A Middle Pleistocene age (MIS8; <270 ka) for the mud is confirmed by the presence of *E. huxleyi* coccolithophores down to the base of the drift, as well as a distinctive nannofossil assemblage^35^ (the complete nannofossil and foram assemblage is summarized in Appendix A). The basal sand sits on a pronounced erosional unconformity, overlying Upper Cretaceous (Campanian-Santonian) mudstone, based on the presence of the dinoflagellate *Isabelidinium cretaceum* and other microfossils^35^. This mid-Pleistocene age is significantly younger than previous published estimates for the SFSD, which inferred an Early Miocene onset of drift deposition, and a series of intra-drift unconformities of Miocene to Pliocene age^18,20^. Our results show that onset of drift deposition was much more recent than any published age for the onset of ACC circulation^13^, and that the intra-drift unconformities must represent fluctuations in current strength on glacial-interglacial, or shorter, timescales.

The LFSD has not been penetrated by any geotechnical boreholes, thus its age remains uncertain. A number of prominent intra-drift unconformities are present (Fig. 2A, inset), which could be tentatively correlated to the unconformities in the SFSD (Fig. 2B), but this remains speculative.

### Falkland Terrace and Sand Sheet

**Figure 3:**
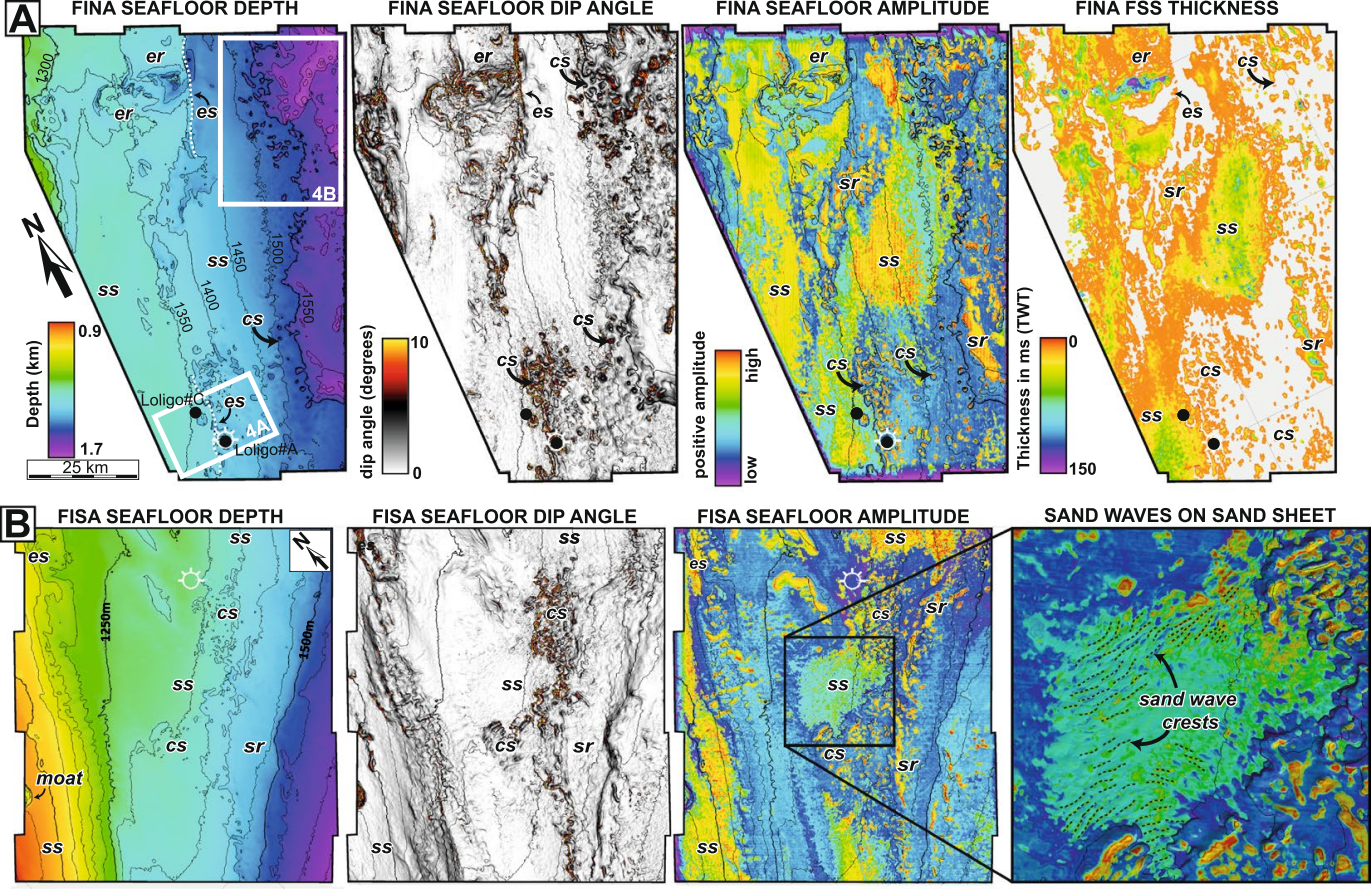
Seismic attributes from 3D data across the Falkland Terrace and Falkland Sand Sheet. Locations of the FINA (**A**) and FISA (**B**) survey are shown in Fig. 1. Attributes include depth, dip angle and absolute amplitude for both surveys, as well as an isopach (sediment thickness) map of the sand sheet for the FINA survey. ‘Warm’ colours (green-red) on the amplitude maps represent sand, which has a higher acoustic impedance than shale near the seafloor. The main geomorphological features annotated on the maps include individual sand sheets (ss), sand ribbons (sr), circular-elliptical scours (cs), erosional remnants (er) and escarpments (es), all of which are produced by bottom currents associated with the Subantarctic Front.

**Figure 4:**
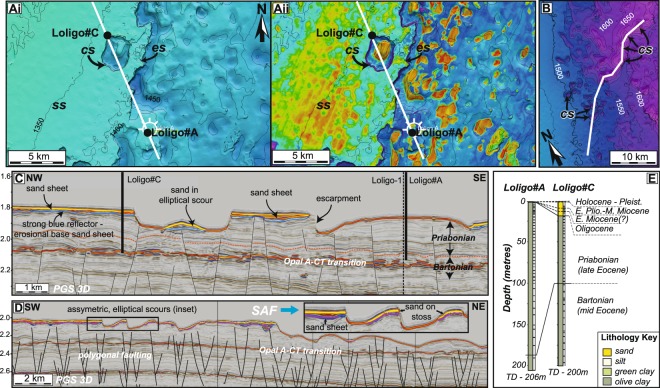
Seismic sections from the FINA 3D survey and geotechnical boreholes through the Falkland Sand Sheet. (**A**) Depth (i) and seismic amplitude map (ii) of the seabed around the Loligo well locations. White line shows location of the seismic section in panel C. (**B**) Depth map of the seabed across a linear array of circular-elliptical scour features in the northeast of the survey area. White line shows location of the seismic section in panel D. Locations of maps A and B are shown in Fig. 3. (**C**) Seismic line across the Loligo#A and Loligo#C geotechnical boreholes. The high-impedance seismic package near the seabed around Loligo#C is absent around Loligo#A. Dashed orange horizon is the Bartonian-Priabonian (37.8 Ma) boundary, which is offset by numerous polygonal faults. (**D**) Seismic line along a linear array of asymmetric circular-elliptical scour features, typically 1–3 km in diameter, and parallel to the main flow of the SAF (blue arrow). Scours are mostly asymmetric, with a steep SW flank and a shallow NE flank, with sand preferentially deposited on the NE (down-current) flank. (**E**) Lithostratigraphic and biostratigraphic correlation of Loligo#C and Loligo#A wells, acquired and analysed by BHP, which penetrate the Falkland Sand Sheet. Core recovery is shown by the black (sample) and white (core gap) barcode to the right of the lithology column. Detailed biostratigraphic data are shown in Appendix B,C.

The Falkland Terrace covers a large area of the eastern Falkland Plateau slope, between 1200 m and 1800 m water depth (Figs 1 and 2C). 3D seismic data across the Falkland Terrace shows that the seabed has a markedly rugose morphology, with numerous erosional features, both parallel and perpendicular to the slope and to modern currents (Fig. 3). These include slope-parallel erosional escarpments, with seabed gradients locally exceeding 45 degrees and relief up to 200 m, plateau-like erosional ‘remnants’ bounded by escarpments, and circular-elliptical scour features, typically 0.5–3 km in diameter and up to 100 m deep (Figs 3 and 4). These scours form in discrete clusters parallel to the slope contours, and are markedly asymmetrical with a steep scarp at the up-current end and a gentler slope extending down-current.

These various erosional features occur both at the seabed and underlying a high-impedance seismic package, which is present across a large area of the Falkland Terrace (Figs 1 and 3). Where present, this high-impedance package has a much higher amplitude seabed reflector than surrounding areas, and a strong negative base reflector (Fig. 4C). This shows that this feature has a significantly higher acoustic impedance than adjacent or subjacent units. This high-impedance package is typically located down-slope of moats (Fig. 2C, inset), on the plateau-like erosional remnants (Fig. 4C), and on the northeast (down-current) side of individual erosional bedforms (Fig. 4D).

Two wells penetrate the shallow stratigraphy, including the high-impedance seismic package. This package is not evident in the seismic data at the Loligo#A location (Fig. 4C). This well penetrated a thin (~2 m, below seismic resolution), sand-dominated interval (Fig. 4E), with a Holocene-Pleistocene age inferred from the presence of *E.huxleyi, C.leptoporus* microfossil assemblages^35^. Neritic foraminifera and the presence of glauconite indicate a shallow water origin for the sediment, whereas abundant Eocene microfossils indicate a substantial degree of reworking of older sediments. Below 3 m, there is a distinct change in both lithology and fossil assemblages, with the sediment being dominated by mudstone of exclusively late Eocene (Priabonian − 35–37 Ma) age.

The Loligo#C well penetrated a thicker (16.5 m) sequence of sand, with fossil assemblages of Oligocene to Holocene age, indicating long-lived coarse-grained sediment transport and reworking. The base of the sand package is most likely earliest Oligocene (~34 Ma), but with a minimum age of 25.1 Ma, based on the abundance of *Chiasmolithus altus* and other Oligocene nannofossil assemblages^35^. Most of the sand is Oligocene-Pliocene in age, with only the shallowest ~1 m of Holocene age. Pleistocene sediments are absent, or were missed due to the sampling frequency. The base of this 16.5 m-thick sand coincides with the base of the high-impedance seismic package, which is evident from the seismic at this location, confirming that the high-impedance seismic package represents an extensive Oligocene-Recent sand deposit, herein referred to as the Falkland Sand Sheet. Below ~16.5 m, there is a distinctive change to latest Eocene – earliest Oligocene mudstone, with a distinct absence of reworked fossil assemblages. Unequivocal Priabonian (late Eocene) species dominate the mudstone sequence from ~26 m depth, with Bartonian (middle Eocene) species occurring below 99 m.

The high-impedance seismic package which we interpret as the Falkland Sand Sheet has been mapped across both the FINA and FISA surveys, as well as on the 2D data. It covers around half of the seafloor across the Falkland Terrace, (Figs 1 and 3), a total area of more than 30,000 km^2^. Where present it is typically 10 ms (minimum seismic resolution) to 75 ms ‘thick’ in TWT (~60 m thickness) and locally as much as 150 ms TWT (~112 m true thickness) (Fig. 3A). Assuming an average thickness, of ~30 m where present (50% of the total area), we estimate the total volume of sediment in the Falkland Sand Sheet to be around 900 km^3^. As such, this is the largest contourite sand sheet to be recognized anywhere in the modern or ancient sedimentary record, to our knowledge.

Extensive individual sand sheets, ‘ribbons’ and sand waves, are evident in both map view and on seismic cross sections. Individual sand sheets range in size from several hundred to over 2000 km^2^ (Fig. 1). Individual sand ‘ribbons’, which may represent sand infilling elongate erosional troughs rather than depositional bedforms, can be traced over at least 2–10 km (Fig. 3). The gently sinuous sand wave crests extend up to 5 km laterally, with wavelengths typically around 500 m, and are oriented perpendicular, or highly oblique, to the isobaths (Fig. 3B). The sand has a complex relationship with the erosional features mentioned above, many of which have evidently pre-dated the deposition of the sand itself, and then partly controlled the location of sand deposition. This is particularly evident within the circular-elliptical scour features, where the sand is preferentially deposited on the shallow-dipping upstream-facing side relative to prevailing currents (Fig. 4). Although these are primarily erosional bedforms, we interpret the dominant process as being analogous to cyclic steps in turbidite systems, where the flow initially accelerates and becomes more turbulent (and erosive) on the lee-side of the scours, and then decelerates and deposits sediment on the stoss side^36^. This indicates that the currents are supercritical, at least periodically, and that flow was continuously south-west to north-east, as in the present day.

It is clear that the whole of the Falkland Terrace has been affected by prolonged and extensive erosion. Such slope-parallel terraces are known to form under the persistent action of bottom currents, especially where the interface between water masses impinges on the seafloor^37^. It is here that kinetic energy transferred to the seafloor is at a maximum. Significant erosion in this case may partly coincide with the interface (pycnocline-thermocline couple) between the AAIW and UCDW, which intersects the slope at around ~1200 m water depth. Hydrodynamic activity at this interface can be elevated by an order of magnitude or more in the presence of benthic storms or by the action of internal waves^38^. However, although the updip limit of the Falkland Terrace coincides with the AAIW-UCDW pycnocline, the erosional features extend well down the continental slope to a depth of 1800 m or more, corresponding closely with the location of the Subantarctic Front^17^.

Although there is strong evidence that the primary mechanism for such pronounced erosion was (and is) strong bottom currents associated with the SAF, measured current velocities at the seafloor in the region of the SAF are only in the order of 0.1–0.2 ms^−1 ^^17^, which are significantly lower than the predicted current strength for generating the erosive (>1 ms^−1^) and depositional (0.5–0.8 ms^−1^) bedforms observed^39^. Erosion may be enhanced by the presence of episodic, fast-flowing, meso-scale eddies and vortices related to the interaction of the front with complex seafloor topography. In the area around the 54–54 passage, such eddies have been observed in present-day seismic oceanographic records^40^, with modelled velocities of several metres per second in the area^1^. However, both eddies and turbulence at the pycnocline would also affect the plateau to the south, in the vicinity of the SFSD, where erosion and coarse-grained deposition has not occurred since the Pleistocene. Therefore, it seems likely that the currents which generated the Falkland Sand Sheet were significantly stronger than those forming the Pleistocene-Recent SFSD.

### Eocene-recent evolution of the subantarctic front

It is evident from our seismic stratigraphic observations and data from the shallow boreholes that substantial changes in SAF intensity have taken place since the Paleogene. Eocene sediments penetrated in Loligo#A and Loligo#C are dominated by biosiliceous pelagic sediment, with little terrigenous input and no evidence of bottom-current reworking of older sediment in the deep ocean. By contrast, the Oligocene-Recent sediment encountered in the Falkland Sand Sheet sits on top of an erosional surface, with evidence from seismic reflection terminations, and our interpretation of an exhumed Opal A-CT transition, for up to 300 m of erosion in places, clearly formed by high-energy along-slope bottom currents. We interpret the unconformity as marking the onset of strong bottom currents associated with the SAF in the Falkland Plateau region. This major oceanographic change occurred after 35 Ma, in the earliest Oligocene (or possibly latest Eocene), and by 25 Ma at the latest. Our interpretation of the data is that the erosional unconformity occurred at or very close to the Eocene-Oligocene boundary.

This erosional unconformity was not only a result of uplift of an active forebulge^34^, but was primarily caused by intense bottom current erosion throughout the region, acting over a long period of time. This, therefore, provides us with a most likely age (~34 Ma) of ACC onset, which precisely matches the generally accepted onset of global cooling at the Eocene-Oligocene boundary. It is consistent with the hypothesis that thermal isolation of Antarctica by the ACC was a fundamental component of this transition, rather than global cooling being purely a function of atmospheric CO_2_ levels^41^. Recent studies have shown a strong coupling between atmospheric CO_2_ and oceanographic processes in the Pleistocene^4^, likely impacted by variations in upwelling from deep CO_2_ laden waters^42,43^ due to changes in wind-strength that drives the ACC^5^. The onset of the wind-driven ACC likely had a direct role in changing atmospheric CO_2_ concentrations at the Eocene-Oligocene transition as well. We note that this predates the onset of ocean spreading in the Drake Passage (~26.5 Ma^22^), and indicates that an intermediate-depth gateway must have formed by the early Oligocene, which was sufficient to allow shallow and intermediate water circulation around Antarctica. These data also necessitate the presence of a sufficiently deep 54–54 passage in the North Scotia Ridge to allow the SAF to flow across the bathymetric barrier by this time. We note that a marked Eocene-Oligocene unconformity is evident elsewhere along the Atlantic margins indicating that the entire AMOC was affected^44^.

The onset of drift deposition in the SFSD and LFSD, by contrast, was significantly later, with a Middle Pleistocene age interpreted for the base of the penetrated sequence. Although the oldest drift sediments have not been penetrated^18^, extrapolation of sedimentation rates from the upper succession would suggest that the entire drift is younger than 0.5–0.7 Ma. Minor erosional unconformities within this are associated with thin silt or sand beds, indicating that they were formed by elevated current conditions, most likely due to the alignment of the SWW with the Drake Passage to the east. This may also explain the onset of sedimentation in the muddy drift at this time, as it seems that current conditions prior to the Pleistocene were likely too strong to allow the deposition of fine-grained sediment. Ongoing erosion at this location continued from the Oligocene until the Pleistocene, by which time weaker current conditions allowed the muddy drift to accumulate. We hypothesize that this decrease in current strength between the Pliocene and the Pleistocene was a result of the northward migration of the SWW, in response to the onset of northern hemisphere glaciation, as has previously been proposed for DO events^11^ and interglacial-glacial transitions^12^.

Given this hypothesis, it is not yet clear why the onset of drift deposition occurred near the mid-Pleistocene, rather than at the end of the Pliocene. We speculate that this may be related to a change in ACC intensity following the middle Pleistocene transition (~0.7–0.9 Ma), possibly due to increasing variability of sea-surface temperatures^45^, or possibly in the transition from ‘lukewarm’ to warm interglacials at 450 ka^46^. However, the long-term trend is clear. The extended period of erosion and coarse-grained sediment deposition from the Oligocene to the Pliocene was due to the onset a prolonged high-energy of bottom currents in the region. The onset of fine-grained (mud) deposition in the Pleistocene was due to a marked slow-down in current speed, suggesting that the latitude and strength of the SWW was closely coupled to the climate state as the Earth entered ice-house conditions. Our data suggests that SWW were stronger and better aligned with the Drake Passage in the Oligocene-Pliocene, when atmospheric CO2 content and global temperatures were substantially higher, driving a faster-flowing ACC than at present, even at intermediate depths.

This is of fundamental importance to future models of ocean-atmosphere interactions in the southern hemisphere, as the SWW are currently moving southwards and intensifying in response to climate change^8^, resulting in an increase in shear stress on surface waters. Our data suggests that this will result in a pronounced strengthening of ACC circulation at intermediate-to-deep depths, as well as of surface currents. The nature and strength of past circulation, therefore, is clearly an important key to predicting future patterns of circulation and their influence on climate. The link between climate state and current strength remains controversial, however, and requires more high-resolution data from contourite deposits around the Southern Ocean, including the SFSD. The results from ongoing scientific drilling expeditions around the Southern Ocean in the coming years, will be crucial to our further understanding of the feedbacks between atmospheric circulation, ocean circulation and climate in a warming world.

## Methods

We use a regional 2D multichannel seismic reflection dataset and two large (total of ~12,000 km^2^) 3D seismic reflection volumes (the FINA and FISA surveys), as well as biostratigraphic and lithological data^35^ from three geotechnical boreholes (Toroa#1, Loligo#A and Loligo#C; Fig. 1), and two deep exploration boreholes (Toroa-1 and Loligo-1), to calibrate stratigraphic age and lithology. Core recovery for the geotechnical boreholes is typically 10% on average. Biostratigraphic data from these boreholes are summarized in the Appendices, and the original data are available from the Falkland Island Government’s Department of Mineral Resources (https://www.fig.gov.fk/minerals/).

The 3D surveys were acquired by PGS using two Bolt LLXT airguns with an air pressure of 2000 psi and 4130 cubic inch volume. Data were recorded using an array of 12*6.6 km long streamers at 120 m spacing, each with 528 Hydroscience 24 bit hydrophones at a 12.5 m group interval. The data were processed using a 3D Kirchhoff pre-stack time migration (PSTM) sequence.

Shallow seismic velocities (*v*) of around 1600–2000 metres per second, and a dominant frequency (*f*) of ~40–50 Hz give a vertical resolution (*v/4f*) of around 10 m for the shallow sections of these surveys, with a horizontal resolution of 12.5 m. Well-to-seismic ties were carried out using an average velocity of 1520 m/s for shallow sediments, derived from calibrated time-depth pairs in the Loligo-1 well (Fig. 1). Seismic interpretation was carried out using Schlumberger Petrel 2015 software, including horizon mapping, dip and amplitude extractions for seismic geomorphological analyses^47^.

## Supplementary information

Dataset 1
