# Supplementary material for: Erosion and deposition beneath the Subantarctic Front since the Early Oligocene
Source: Sci Rep. 2019 Jun 26;9:9296. doi: 10.1038/s41598-019-45815-7 (PMC6594945; doi:10.1038/s41598-019-45815-7)
Supplement: Supplementary file 1 — Dataset 1 [file 41598_2019_45815_MOESM1_ESM.pdf]

Erosion and deposition beneath the Subantarctic Front since the Early Oligocene

Uisdean Nicholson[1*] Dorrik Stow[1]

[1]*School of Energy, Geoscience, Infrastructure and Society, Heriot-Watt University, Edinburgh EH14 4AS, Scotland, UK.*

**Correspondence to* *u.nicholson@hw.ac.uk*

Appendices: Biostratigraphic data from geotechnical boreholes on the Falkland Plateau

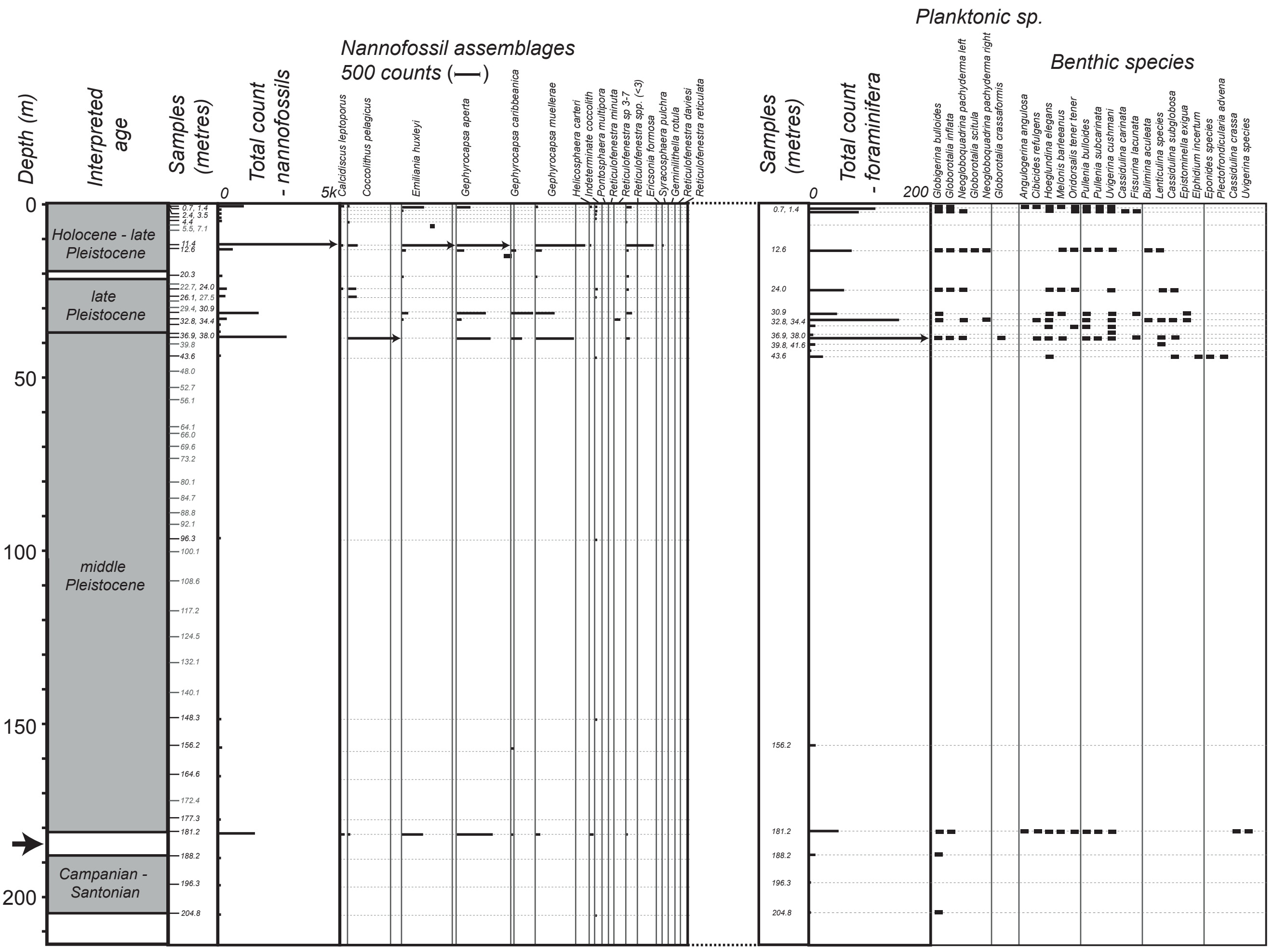

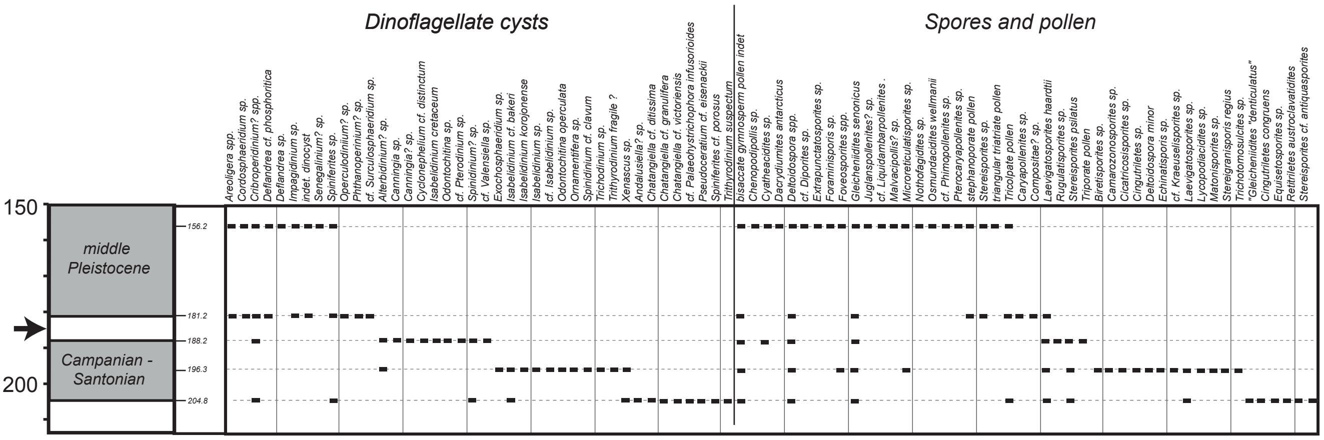

Basis for age interpretation

Holocene - late Pleisotene interpreted on the basis of the common occurence of *Emiliana Huxleyi* and *Gephyrocapsa aperta, as well as C. leptoporus, H. Carteri* and *C. Pelagicus*. The abundance of *Emiliana Huxleyi* (up to 50% os samples) suggests a Late Pleistocene (<70ka) or younger age, when this species bacmae dominant. Assemblages are consistent with those from interglacial conditions.

Late Pleistocene interpreted on the basis of the increase of *C. Pelagicus* (more common during glacial periods) over other species, with assemblages otherwise similar to above.

Middle Pleistocene interpreted on the basis of the presence of *Emiliana Huxleyi, G. Aperta, and C. Pelagicus* nannofossil species, with the relative abundance of these species indicating fluctuating cold (glacial) and warm (interglacial) conditions. *E. Huxleyi* constrains the maximum age to ~290ka. Reworked Eocene dinoflagellates are also present.

Campanian - Santonian interpreted on the basis of the presence of the dinoflagellates *Isabelidinium Cretaceum and* Trithyrodinium suspectum, as well as Upper Cretaceous spores. The samples are barren of nannofossils and foraminifera.

Thick black arrow shows the location of the base of the base-drift unconformity

Appendix A: Summary of nannofossil and foraminifera assemblages (top) from Toroa#1, as well as dinoflagellate, spore and pollen assemblages from the bottom ~50m of the well. Modified from Crux (2009)

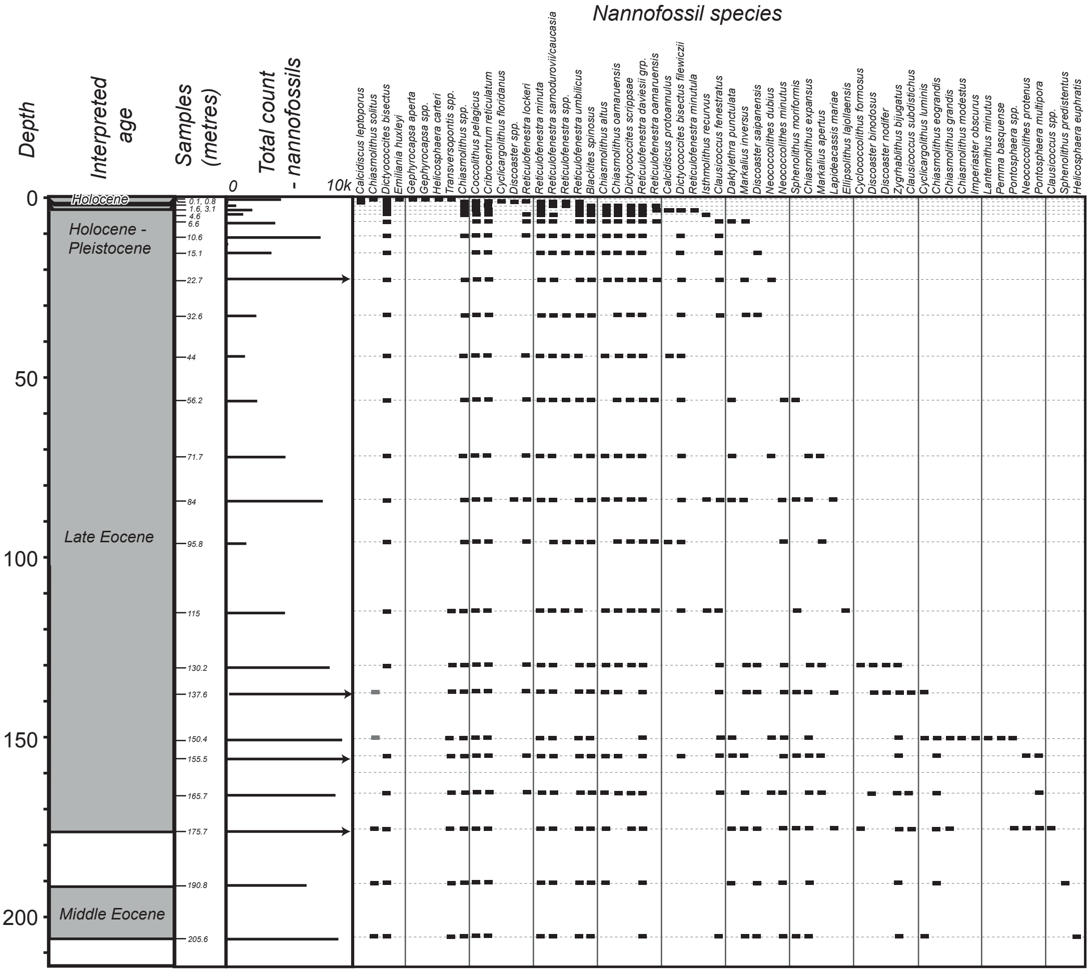

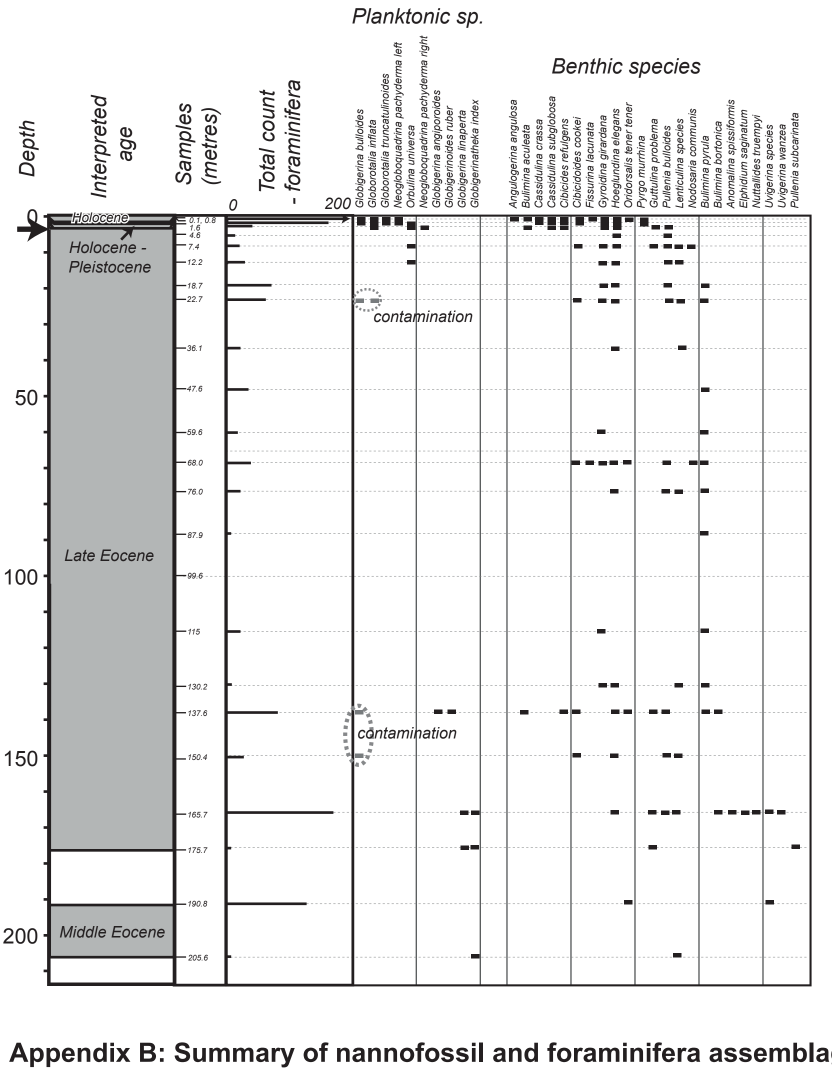

Basis for age interpretation

Holocene interpreted on the basis of the dominance of *Emiliana Huxleyi* and the warm water species *C. leptoporus*. Could be as old as middle Pleistocene. Abudant reworked Eocene fossils

Holocene-Pleistocene interpreted on the basis of *Globigerina bulloides, Globorotalia inflata* and *Neoglobigerina pachyderma.* Nannofossils are exclusively reworked Eocene species.

Late Eocene (Priabonian) interpreted on the basis of numerous Eocene species, including *Daktylethra punctulata* and *Discoaster saipanensis*. Contamination is interpreted between depths of 7.4-22.7 m and 137.6-150.4 m (grey samples).

Middle Eocene (Bartonian) interpreted on the basis of the first downhole appearance of *Chiasmolithus solitus*. The presence of *C. reticulatum* and *Globigerinatheka index* indicates an age no older than 42 Ma.

Thick black arrow shows the location of the base of the Falkland Sand Sheet

Appendix B: Summary of nannofossil and foraminifera assemblages from Loligo#A, modified from Crux (2009)

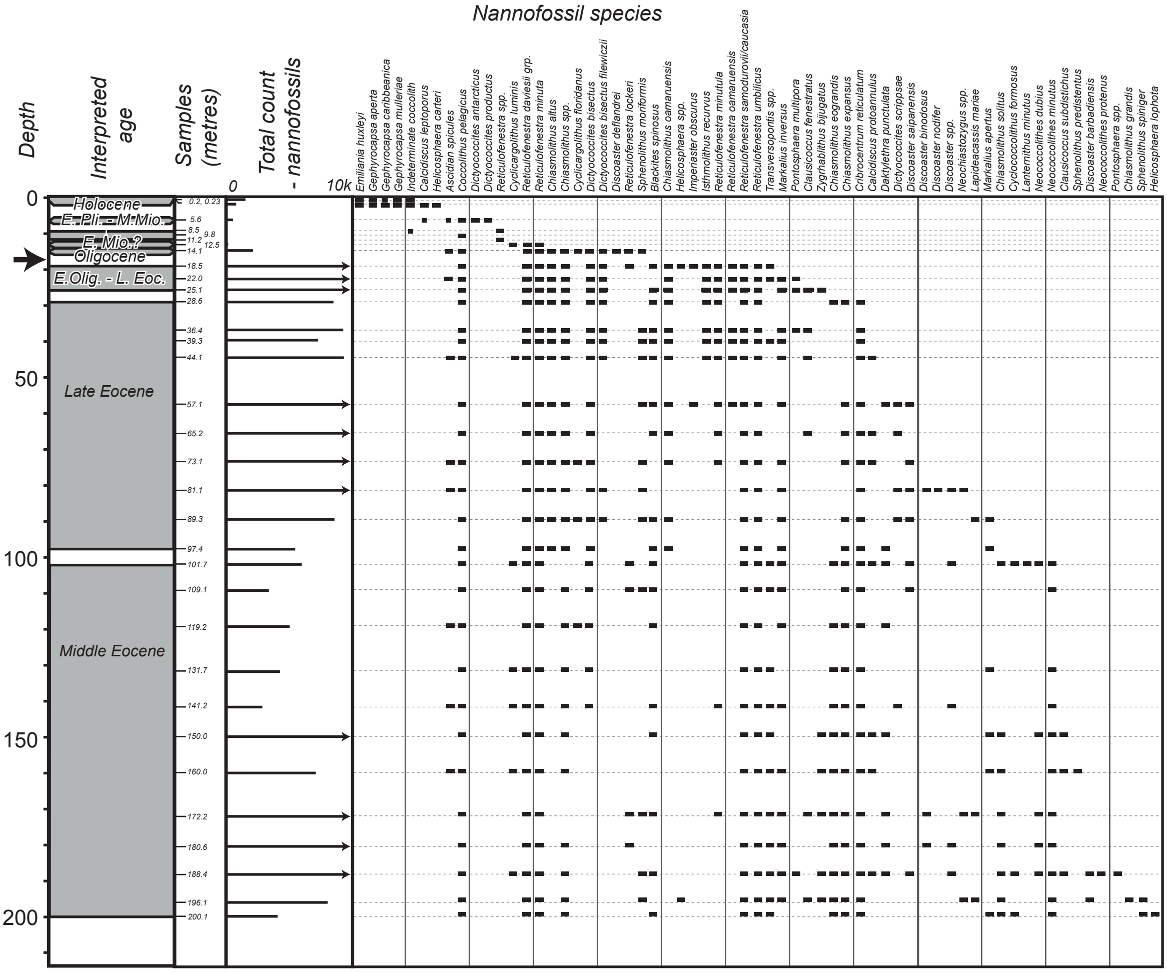

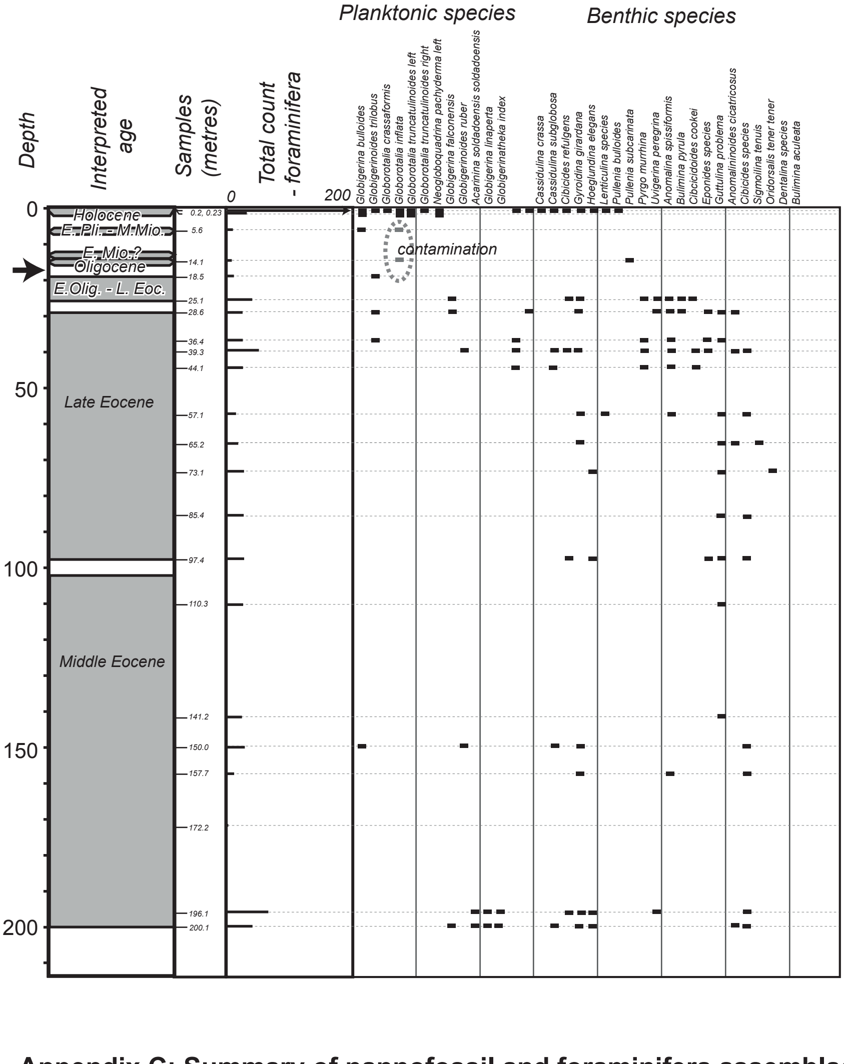

Basis for age interpretation

Holocene interpreted on the basis of the dominance of *Emiliana Huxleyi* and the common occurence of *Gephyrocapsa aperta*, *G. caribbeanica*, and *G. mulleriae. leptoporus* and the warm water species *C. leptoporus*. Could be as old as middle Pleistocene. Foram species including *Globigerina bulloides, Globorotalia inflata, G. truncatulinoides and sinistrally coiled Neogloboquadrina pachyderma* are also consistent with a Holocene age

Early Pliocene - Middle Miocene interpreted on the basis of *D. antarcticus. G. inflata* considered to be contamination. No benthic forams reported.

Early Miocene (or older) interpreted on the basis of the *Reticulofenestra daviesii* group. No samples examined for foraminifera

Oligocene interpreted on the basis of the common occurence of *Dictyococcites bisectus, D. bisectus filewiczii, Chiasmolithus altus* and abundant *Cyclicargolithus floridanus*. Late Oligocene or younger *Cyclicargolithus abisectus* is absent.

earliest Oligocene-latest Eocene interpreted based on the first downhole occurence of *Reticulofenestra oamaruensis, R. umbilicus, Isthmolithus recurvus* and *Chiasmolithus oamaruensis*, and the absence of Late Eocene species recorded below

Late Eocene and **Middle Eocene** ages assigned as for Loligo #A (Appendix B).

Thick black arrow shows the location of the base of the Falkland Sand Sheet

Appendix C: Summary of nannofossil and foraminifera assemblages from Loligo#C, modified from Crux (2009)
